# Keloid transcriptomics reveal heterogeneity in fibroblast subtype enrichment, gene expression, and immune cell responses

**DOI:** 10.64898/2026.09.02.747090

**Published:** 2026-09-07

**Authors:** Jonathan J. Panzer, Mingming Pan, Mrudula Nair, Ian M. Loveless, Xiangguo Dai, Indra Adrianto, Ling Huang, Dhananjay Chitale, Ralph Francescone, Debora B. Vendramini-Costa, Cristina de Guzman Strong, Albert M. Levin, Lamont R. Jones

## Abstract

Keloid disease (KD) is a fibroproliferative skin disorder resulting from abnormal scar formation that causes pain, itching, and decreased quality of life. While multiple KD transcriptomic studies exist, the influence of cell type composition on bulk tissue gene expression is unknown. We characterized fibroblast subtype and immune cell enrichment using bulk RNA-Seq of head and neck keloid and matched adjacent normal skin tissue (MANST) from 14 patients (10 African American and 4 European American). Cell type enrichment was calculated by single sample gene set enrichment analysis. Linear mixed-effects models were employed for 1) differential cell type enrichment across tissue, 2) tissue type-specific associations between fibroblast subtypes and immune cells, and 3) differentially expressed genes (DEGs) across tissue. Validation was conducted in an independent cohort of 8 African Americans. Three fibroblast subtypes and 14 immune cell types were differentially enriched across tissue type. Further, 17 tissue type-specific fibroblast subtype-immune cell enrichment associations were identified, with 14 exhibiting decreased association in keloid tissue relative to MANST. After adjustment for cell type enrichment, MIR31HG and NR4A2 were significant DEGs with the largest positive and negative fold-changes, respectively. By considering cell type enrichment, underlying keloid tissue-specific cell type and gene expression associations were revealed.

## Full Text Availability

The license terms selected by the author(s) for this preprint version do not permit archiving in PMC. The full text is available from the preprint server.

